# Parenthood—A Contributing Factor to Childhood Obesity

**DOI:** 10.3390/ijerph7072800

**Published:** 2010-06-30

**Authors:** Fatma G. Huffman, Sankarabharan Kanikireddy, Manthan Patel

**Affiliations:** Department of Dietetics and Nutrition, Robert Stempel College of Public Health and Social Work, Florida International University, 11200 SW 8th St., HLS1-435, Miami, FL 33199, USA; E-Mails: Skani@fiu.edu (S.K.); Manthan.Patel@fiu.edu (M.P.)

**Keywords:** children’s diet, childhood obesity, NHANES, single-parent households, BMI, blood-cholesterol

## Abstract

Prevalence of childhood obesity and its complications have increased world-wide. Parental status may be associated with children’s health outcomes including their eating habits, body weight and blood cholesterol. The National Health and Nutrition Examination Survey (NHANES) for the years 1988–1994, provided a unique opportunity for matching parents to children enabling analyses of joint demographics, racial differences and health indicators. Specifically, the NHANES III data, 1988–1994, of 219 households with single-parents and 780 dual-parent households were analyzed as predictors for primary outcome variables of children’s Body Mass Index (BMI), dietary nutrient intakes and blood cholesterol. Children of single-parent households were significantly (p < 0.01) more overweight than children of dual-parent households. Total calorie and saturated fatty acid intakes were higher among children of single-parent households than dual-parent households (p < 0.05). On average, Black children were more overweight (p < 0.04) than children of other races. The study results implied a strong relationship between single-parent status and excess weight in children. Further studies are needed to explore the dynamics of single-parent households and its influence on childhood diet and obesity. Parental involvement in the development of school- and community-based obesity prevention programs are suggested for effective health initiatives. Economic constraints and cultural preferences may be communicated directly by family involvement in these much needed public health programs.

## 1. Introduction

According to the 2003–2004 National Health and Nutrition Examination Survey (NHANES) the percentage of overweight children increased from 6.5% in 1980 to 17.1% in 2004, placing childhood obesity as one of the major lifestyle concerns of the United States [1,2]. Although the prevalence of overweight children and adolescents of all ages (2–19 years) is on the rise, elementary school aged children (ages 6–11 years) have the highest prevalence of overweight (18.8%) [1]. Overweight in children is determined by plotting a child’s Body Mass Index (BMI) on a Center for Disease Control (CDC) growth chart; children whose BMI is greater than the 95th percentile as compared to children of the same sex and age are considered obese [1]. Currently, the CDC classifies a child as obese at or above the 95th percentile of weight for their age and gender and as overweight if the child is at or above the 85th percentile [3]. Although not all obese infants become overweight children, and not all overweight children become obese adults, there is a greater likelihood that obesity beginning in early childhood will persist throughout the life span [4]. In addition to increasing the risk of obesity in adulthood, childhood overweight is also the leading cause of pediatric hypertension and is associated with type 2 diabetes mellitus [5]. Obesity also increases the risk of coronary heart disease, places extra stress on the weight-bearing joints, and is associated with high incidence of liver disease and asthma [6]. Besides these physical effects, being overweight may have deleterious psychological effects on children such as: lowering self-esteem; affecting relationships with peers; and, causing social problems [7].

Etiologies of childhood overweight include modifiable and non-modifiable risk factors. Modifiable causes include physical inactivity, sedentary life style, low socioeconomic status, unhealthy eating habits, and environmental factors [8]. Specifically, modifiable risk factors for children include lack of regular exercise, high frequency of television viewing or computer usage, low family income, non-working parents, over-consumption of high-calorie foods, snacking while watching television or doing homework, and over-exposure to advertisement of high calorie foods. The common non-modifiable cause of obesity is genetics, with greater risk of obesity found in children of obese and overweight parents [9]. Interventions for reducing childhood obesity aimed at modifiable risk factors have been extensively indicated in the literature as key to reversing the detrimental health effects that impact the quality of life in children [11].

It is well documented that the number of single-parent households in the United States is rising. Single-mother families have increased from 3 million in 1970 to 10 million in 2003 (12% to 36%), while single-father families increased from less than half a million to 2 million (1% to 6%) within the same time period [11]. One explanation for the increase in single-parent families may be the rise in late marriages, which increases the likelihood of non-marital births, and is also associated with increased divorce rates. The proportion of single-parent homes differ by race and gender whereby 47% of Black females as compared to 14.4% of White non-Hispanic females and 8.6% Black males as opposed to 5.1 % of White, non-Hispanic males reported being the head of a single-parent household in 2003 [11]. Single-parent households comprised 26% of all households with 76% reportedly headed by women in 2003 [11].

There are significant social problems for children and parents associated with single-parent households; these problems include difficulties with role identity for children and social stigma associated with single-parent status. Single parents often experience role strain when balancing the role of wage earner with parental responsibilities. Research has indicated that children of single parents are less likely to eat at the table together and are permitted to play and watch television during meals [12]. Poverty has been consistently linked with single-parent households, especially those headed by women [13]. Children of female-headed households consumed more total fat, saturated fat and sweetened beverages as well as had a higher percent of “more than two hours of television/video viewing” than children of dual family households [10].

### 

#### Objectives

The primary objective of this study was to evaluate the association between parental status and childhood overweight. Secondary objectives were to assess dietary and blood cholesterol differences of children from single or dual households. We hypothesized that in a national population there would be higher obesity rates for children from single-parent households than for children of dual-parent households and that children of single-parent households would consume more calories from fat than children from dual-family households.

## 2. Methodology

### 2.1. Sample

The NHANES III data-set was appropriate for the study since it facilitated matching children with their parent’s data. The NHANES survey design is a stratified, multistage probability sample of the civilian non-institutionalized U.S. population. Data were extracted from NHANES III during the time period 1988–1994, matching parents with children and forming the dataset: Resier I A Files. The respondents’ included 1,000 children aged 6–11 years from 219 households with single-parents and 780 with dual-parents. The continuous physical variables analyzed for the children were age, sex, race, height, weight, and BMI. Dietary recall data were collected and included the following: consumption of total energy; carbohydrate; protein; total fat; saturated fat; and, mono/poly unsaturated fatty acids. Single dietary recalls were obtained from children but proxy was used by care providers and from the school if the children were unable to answer the questions. Children were matched to their corresponding parents based on family sequence numbers in the adult and youth data files. The variables analyzed for the parents were sex, marital status, level of education, and annual family income. Physical activity of children was not measured by the survey and could not be estimated from the data. Data acquisition of parenthood status by NHANES III allowed us to classify the children as living in a single-parent household if widowed, separated and divorced caretakers were reported. Matching of parents and children was not possible in previous years and has not been available in subsequent surveys. All other children were considered to be living in dual-parent households.

### 2.2. Anthropometrics

A systematic, randomly selected, sub-sample from the general NHANES sample was asked to participate in a more detailed data collection including: measurements of body weight and height; fasted blood draw to assess blood cholesterol levels; and, a dietary assessment. An appointment was set for health measurements at a local mobile center with trained medical personnel and childcare services were provided during the participants’ examination and interview. Respondents agreed to and signed an additional informed consent form. The details of the procedure are available at the NHANES website. There are slight variations each year; however, the general procedures are consistent.

Though BMI is calculated as weight (kg)/height (m^2^) in adults, a different method is applied for children, which further classifies them based on age and sex. According to the CDC definition, children whose BMI is greater than the 95th percentile are considered obese and children whose BMI range between the 85th and 95th percentile are classified as overweight [3]. Children with BMI between the 5th and 85th percentile are categorized as normal weight and children less than the 5th percentile are considered underweight. The criteria for weight status of different age groups are given in Table 1.

### 2.3. Dietary Assessment Methods

The detailed dietary recall method used by NHANES was a 24 hour recall. Trained interviewers using computer-assisted dietary software asked participants (in English or Spanish) questions regarding what they ate and drank for a complete day from midnight to midnight. Translators were available and questions could be answered by proxy. Visual aids, charts and graphs were used by the participant to recall the type or brand of food/beverage and the quantity. The interviewer recorded the conversation for transcription purposes and the process usually took between 15 and 30 minutes. An additional 10% of participants were telephoned for an additional 24 hour recall interview either in person or by telephone to improve validity. From the 24 hour recalls, total calorie, protein, carbohydrate and fat consumed per day per person could be assessed.

### 2.4. Primary Outcome Variable

For our study, the variable, BMI was chosen as the primary indicator of obesity because it is considered an appropriate indicator of body fatness. A binary variable for obesity (95th percentile) *versus* other was computed for logistic regression models with single parent as the primary predictor and age, parental education, family annual income and race as covariates.

### 2.5. Statistical Analysis

Descriptive statistics were calculated to determine the percentage of overweight children by sex and parental status. The two groups were normally distributed and had approximately equal variances of their Body Mass Index which was verified by Levine’s test. The two groups were independent of each other. The association of parental status and childhood overweight was evaluated by comparing mean BMI, dietary recall data, and blood parameters between the children of single- and dual-parent households. Statistical analyses were performed with independent sample t-tests of the two groups, comparing the BMI and dietary recall data among the children living in single- and dual-parent households. Statistical analysis was performed using an independent sample t-test to compare the means of variables including: BMI; dietary intake; and, blood parameters among the various age groups for children of single- and dual-parent households. A one-way analysis of variance (ANOVA) was performed to compare the BMI status of children of different races for single-parent households. To test the association of obesity and parental status, logistic regression models were run with obesity *versus* no obesity as the dependent variable, single-parent household as the independent variable and age, income race and education as the covariates. The final sampling weight and Taylor’s linearization methods were used for variance estimation to account for multistage stratified cluster design. Statistical analyses were conducted using Stata 10.0 SE and the Strata database was imported into SPSS version 18 for the logistic regression analyses. For all analyses, p < 0.05 was considered significant.

## 3. Results and Discussion

Children of single-parent (N = 219) and dual-parent (N = 780) households were almost equally distributed by sex. However, the proportion of overweight children from single-parent households (41%) was more than children from dual parent households (31%) (Table 2).

The social, biological and dietary factors are shown in Table 3. Children from dual parent households had significantly (p < 0.01) lower BMI (19.2 ± 5.4) than children of single-parent households (21.5 ± 6.5). Total caloric intake was marginally significant (p < 0.06) between children of single-parent households (1910 ± 24) and dual-parent households (1,860 ± 25). Mean LDL level was also higher in children of single-parent household (91 ± 1.5) compare to children of dual parent household (88 ± 1.4; p < 0.05). Total fat and saturated fat intakes (g/day) were higher (p < 0.05) in children of single-parent households (9.1 ± 6.2; 3.3 ± 1.0) than children of dual-parent households (8.6 ± 5.2; 3.2 ± 1.1).

Black children tended to be more overweight and had higher (p < 0.04) BMI (20.4 ± 2.2) than White children (19.2 ± 2.2) and children of other races (18.9 ± 3) (Table 4).

The greater likelihood (OR: 1.72 (1.24, 2.38), p = 0.001) of being obese from a single-parent household was confirmed by binary logistic regression. Model 1 tested the likelihood of being a single parent with child obesity. Model 2 included child’s age and parental education level and was the best model. Model 3 added race and income as covariates and was not better than model two (Table 5).

### 3.1. Discussion

This study found that children from single-parent households tend to be more overweight than children from dual-parent households. These results are in opposition to an obesity study of matched children and parents (N = 600 children) randomly selected from two community and two hospital clinics in New Haven, CT [14]. The investigators found lower obesity rate for children living with grandparents or in foster care than for either dual- or single-household parents [14]. However, the results of our study are in accordance with an earlier epidemiological study of the American family and childhood obesity trends [15], a home environment and childhood obesity national longitudinal study [16] as well as population trends from the United States Census as reported from the US Department of Health and Human Services (DHHS) and several other studies [17–20].

Numerous studies regarding parental factors such as hours of maternal employment and parents’ BMI influencing childhood obesity indicated dietary intake was a confounder and should be considered in future studies. Our results indicated a higher total fat intake for children from single-parent households as opposed to those from dual family households. The results of a consumer expenditure survey conducted by the Bureau of Labor Statistics of approximately 7,500 households indicated that fewer vegetables were purchased by single-parent households (single father or mother) as compared to dual-parent households [20]. Although not causal, these findings suggest a likelihood of higher consumption of dietary fat by children of single- as opposed to dual-parent households since there has been an established negative association of vegetable intake with dietary fat intake [20]. Despite the established positive association of dietary fat and obesity, not all children are diagnosed or treated and those who are treated often receive general dietary advice rather than family counseling aimed at developing appropriate strategies [14].

Our findings of significantly higher cholesterol levels and lower HDL (higher LDL) levels in children from single-parent households may have public health implications. According to current dietary guidelines for children and adolescents, the association between hyperlipidemia and childhood obesity has been well-established [21]. Elevated LDL levels in children, even in the absence of obesity, place them at elevated risk for CVD, hypertension and type 2 diabetes [5,6,21]. Obesity and high blood cholesterol may be the outcome of increased consumption of packaged and convenience foods by single-parent or dual-working parent households [22].

Our results indicate that Black children from single-parent households had significantly higher BMI as compared to White children. Federal reports indicate that the ratio of overweight children ages 6–11 years old in 2003 was significantly higher for African American girls (1.6) as compared to non-Hispanic White girls [23,24]. During 2003–2004 Black females ages 6–17 years were at a 25% greater risk of being overweight than non-Hispanic White females [23].

### 3.2. Strengths and Limitations

A major strength of this study was that the subjects were randomly selected through a national data set which identified persons by race and matched parents with children. The sample size was considerably large and represents all children in United States. There has been an increase in the number of single-parent households in the United States since this study data were collected (1988–1994). Incidence of obesity and overweight has also been increasing exponentially among children. Subsequent NHANES datasets do not include enough information to match children with their parents in order that single-parent or dual parent status can be determined. Therefore, our study is a timely analysis of parental household factors in childhood obesity using the most current national data matching children and parents.

Several limitations of this study need to be noted. First, the study was a cross-sectional analysis and does not establish the temporal relationship between parenthood status and obesity in childhood; hence, causal relationships cannot be established. The dataset did not allow for analysis of single-parent households headed by gender; however, based on census statistics, most single-parent households are reportedly headed by females (76%) [11]. On the other hand, there is data indicating that single-parent households headed by males have been increasing in recent years, 26% of all single-family households were headed by males [11]. Since food allocations differed by gender with respect to single-parent homes [20], it would be of interest to study whether or not there would be any dietary or clinical differences, between the children from single-parent households headed by men *versus* women. Another potential limitation was that the data did not include information about the length of time since divorce or separation which could play an important role in the development of obesity. Although dietary recall was conducted with a trained interviewer and with a standardized protocol, the information was self-reported and may have subject-bias. We did not account for the effect of genetics and physical activity which are associated with obesity among children. Mexican Americans were distinguished from other ethnicities only for certain variables and Hispanics could have chosen to classify themselves as either Black or White. Physical activity was not measured by NHANES for the dataset of matched parents with children. This is perhaps the most serious limitation of the study. Physical activity level is a major component of energy expenditure and is strongly associated with obesity.

## 4. Conclusions

Our results indicate a positive relationship between single-parent status and excess weight in children. There were racial differences in body weight (Black children had higher BMI than White children); however, low family income was not significantly associated with children being overweight in a single-parent to dual parent household-comparison. Children from single-parent households consumed more calories and fat than children from dual-parent households. Blood cholesterol was positively associated with BMI and both are risk factors for cardiovascular disease. Our findings suggest single-parent households may have cultural and social factors contributing to excess weight gain and cardiovascular disease risk in children. Since single-parent households are increasing, further studies are needed to explore the dynamics of single-parent households and its influence on childhood obesity. Despite the fact that parents are central to successful community and school based obesity interventions, they have not been included in the development or design of these programs [22]. Interventions in the schools involving parents and children in the preparation of convenient and healthy meals taking into consideration household status, economic constraints and cultural preferences are warranted.

## Figures and Tables

**Table 1:** BMI corresponding to different age groups, sex, and BMI Percentile (%).

	**BMI%**					
**Age (years)**	**<85%**		**85–95%**		**>95%**	
	
	Male	Female	Male	Female	Male	Female
6–6.5	<16.5	<16.1	16.5–17.8	16.1–17.2	>17.8	>17.2
6.6–7.5	<17.3	<17.2	17.3–19.0	17.2–18.9	>19.0	>18.9
7.6–8.5	<18.1	<18.2	18.1–20.2	18.2–20.4	>20.2	>20.4
8.6–9.5	<18.9	<19.2	18.9–21.5	19.2–21.8	>21.5	>21.8
9.6–10.5	<19.7	<20.2	19.7–20.5	20.2–23.0	>20.5	>23.0
10.6–11	<22.3	<21.2	22.3–23.9	21.2–24.6	>23.9	>24.6

* BMI number is plotted on the CDC BMI-for-age growth charts (for either girls or boys) to obtain a percentile ranking.

**Table 2:** BMI categories of males and females from single- and dual-parent households.

	**< 85% n (%)**		**85–95% Overweight n (%)**		**>95% Obese n (%)**		**Total N = 1,000**	
	
	Male	Female	Male	Female	Male	Female	Male	Female
**Dual Parents**	231 (56.7)	219 (58.7)	47 (11.6)	44 (11.8)	129 (31.7)	110 (29.5)	407	373
**Single Parent**	60 (52.2)	43 (41.3)	13 (11.3)	15 (14.4)	43 (37.4)	47 (44.2)	115	105
**P-value**	0.18	0.81	0.06	**0.02**	**<0.01**	**<0.01**		

**Table 3:** Social, biological and dietary characteristics for single- and dual-parent children.

	**Dual Parent**	**Single Parent**	**P-value**

	**N (%) or Mean ± SD**		
**Children’s Demographic Characteristics**			
Gender			
▪ Male	408	115	
▪ Female	371	103	0.921
Age yrs (8.7 ± 1.5)	8.68	8.73	0.726
Race			
▪ Whites (Hispanics and Non-Hispanics)	467	78	
▪ Blacks (Hispanics and Non-Hispanics	290	136	
▪ Others	22	4	**<0.001**
Annual Family Income			
▪ $9,999 or less	78	62	
▪ $10,000–19,999	166	68	
▪ $20,000–50,000	235	55	
▪ $50,000 or more	257	19	**<0.001**
Parents’ Education Level			
▪ Less than HS or GED	193	74	
▪ HS	289	77	
▪ Some College	143	43	
▪ College degree higher	144	23	**0.007**
**Children’s Biometrics**			
BMI kg/m^2^	19.2 ± 5.4	21.5 ± 6.5	**<0.01**
Total Energy intake Kcal/d	1,860 ± 25.0	1,910± 24.0	**0.059**
Mean Blood Cholesterol mg/dl	158 ± 4.3	159 ± 4.7	0.12
Mean LDL levels mg/dl	88 ± 1.4	91 ± 1.5	**0.04**
Mean HDL levels mg/dl	45 ± 1.2	45 ± 1.1	0.51
**Children’s Dietary Intake (grams/day)**			
Total Protein	9.4 ± 10.7	8.4 ± 10.6	0.20
Total carbohydrate	27.4 ± 4.1	24.8 ± 3.2	0.13
Total fat	8.6 ± 5.1	9.1 ± 6.2	**0.04**
Total Mono Unsaturated Fatty Acid	3.4 ± 1.2	3.4 ± 1.2	0.88
Total Poly Unsaturated Fatty Acid	1.9 ± 0.4	1.5 ± 0.7	0.69
Total Saturated Fatty Acid	3.2 ± 1.1	3.3 ± 1.0	**0.04**

**Table 4:** Mean BMI distribution of children by race.

**Race**	**N = 1000**	**BMI** **Mean ± S.D.**
Whites (Hispanics and Non Hispanics)	548	19.2 ± 2.1^b^
Blacks (Hispanics and Non Hispanics)	426	20.4 ± 2.2^a^
Others*	26	18.9 ± 3.0^b^
P value (ANOVA)		0.04

a, significant at p < 0.05;

b significant at p < 0.001.

* Note: Others are children who did not classify themselves as Black or White and may include Asian/Pacific Islanders, American Indians or Hispanics.

**Table 5:** Odds ratio of obesity for a single-parent household.

**Independent Variables**	**Model 1** Step Coefficients:χ^2^(1df) = 10.3, p = 0.001			
Marital Status (Single)	Beta	SE	OR	P
	0.540	0.167	1.72 (1.24, 2.38)	0.001

	**Model 2** Step Coefficients:χ^2^(4df) = 38.9, p < 0.001			
Parental Education	Beta	SE	OR	P
	-	-	Categorical (3df)	<0.001
Age	−0.016	0.004	0.984 (0.976, 0.992)	<0.001

	**Model 3** Step Coefficients:χ^2^(6) = 7.22, p = 0.301			
Annual Family Income	Beta	SE	OR	P
	-	-	Categorical (4df)	0.148
Race	-	-	Categorical (2df)	0.802
